# Physiological Indicators of Acute and Chronic Stress in Securely and Insecurely Attached Dogs Undergoing a Strange Situation Procedure (SSP): Preliminary Results

**DOI:** 10.3390/vetsci9100519

**Published:** 2022-09-23

**Authors:** Giacomo Riggio, Carmen Borrelli, Marco Campera, Angelo Gazzano, Chiara Mariti

**Affiliations:** 1 Department of Veterinary Sciences, University of Pisa, 56124 Pisa, Italy; 2 Department of Biological and Medical Sciences, Oxford Brookes University, Oxford OX3 0BP, UK

**Keywords:** cortisol, blood pressure, heart rate, saliva, bond, attachment, dog, stress, Ainsworth test

## Abstract

**Simple Summary:**

The attachment bond that dogs form towards their owners shares similar features with the bond children form towards their caregivers. Insecurely attached children struggle to find support from their caregivers and therefore to regulate their own emotional response in times of distress. We aimed to investigate whether the quality of dog attachment to the owner may affect their physiological response to stress. We selected ten insecure and ten secure dogs from a sample of individuals who underwent a Strange Situation Procedure (SSP) to assess their attachment pattern towards the owner. The SSP is specifically designed to progressively generate stress. We collected saliva samples before and after the test to measure cortisol concentrations, as an indicator of acute stress, as well as a hair sample to assess chronic stress. We also measured blood pressure, heart rate, respiratory rate and rectal temperature after the completion of the test. The results showed that salivary cortisol concentrations were higher in insecure dogs, particularly after the test. Heart rate also tended to be higher in insecure dogs. No difference in hair cortisol levels were found between secure and insecure dogs. Dogs’ physiological response to acute stress may be affected by the quality of the attachment to the owners.

**Abstract:**

The quality of the attachment bond towards the caregiver may affect the dog’s physiological responses to stressful stimuli. This study aimed to measure chronic and acute physiological parameters of stress in ten securely and ten insecurely attached dogs. The twenty experimental subjects were selected from a sample of dogs that participated with their owners in the Strange Situation Procedure. Saliva samples were collected before (T0) and after (T1) the test. Blood pressure, heart rate, respiratory rate, and rectal temperature were measured after the test, only. At this time, a hair sample was also collected. RM ANOVA was used to analyse cortisol concentrations between secure and insecure dogs at T0 and T1. Mann–Whitney U test or T test were used for other physiological parameters. Insecure dogs had significant higher salivary cortisol concentrations than secure dogs at T1 (*p* = 0.024), but only a non-significant trend towards higher cortisol concentrations at T0 (*p* = 0.099). Post-test heart rate also tended to be higher in insecure compared to secure dogs (*p* = 0.077). No significant differences in hair cortisol concentration were found. The quality of attachment may affect the dog’s physiological response to acute stress, at least when related to separation from the caregiver. The effect of attachment on chronic stress requires further investigation.

## 1. Introduction

The term “attachment” was originally introduced by Bowlby [1] to describe a specific type of affectional bond that a human infant forms between himself and the mother, or another specific individual that acts as a caregiver [2]. The pivotal concept of attachment, as well as its purpose in evolutionary terms, is the infant seeking for care and protection from the caregiver, through a set of behaviours aimed at maintaining proximity and/or contact [3]. The way the caregiver responds to the infant’s attachment behaviour affects the quality of the attachment bond and shapes the infant’s perception of himself and others in the context of future social relationships [4].

More recently, the attachment construct has been applied to relationships between individuals of other mammal species, such as canids [5,6,7,8] and primates [9], as well as to those between members of two different species, such as dogs and their owners [10,11,12,13,14,15,16,17,18]. This is because the dog-owner relationship appears to show similar features with the child-caregiver bond. In order to investigate those features, anthrozoology researchers used modified versions of the same laboratory test used to assess the quality of the child-caregiver attachment bond, the Strange Situation Procedure (SSP) [19]. The SSP is a 20-min long laboratory procedure aimed at progressively increasing stress in the tested individual, and therefore activating his attachment behavioural system towards the caregiver [20]. This occurs through repeated separations from and reunions with the caregiver and from meeting a stranger while in an unfamiliar environment [20]. Just like in children, the SSP allowed to recognize four patterns of dog’s attachment behaviour towards the owner, namely secure, insecure-avoidant, insecure ambivalent and insecure-disorganized [21,22]. The three insecure patterns can be grouped into one broader insecure category, as often reported in the scientific literature [21,22,23]. In human psychology, it is well recognized that securely attached individuals have caregivers who consistently respond in a sensitive and supportive way to their needs [24]. On the contrary, insecurely attached individuals have caregivers who are either rejecting, inconsistent or even abusive and/or neglectful in their responses, leading to different patterns of insecure attachment, that is insecure-avoidant, insecure ambivalent and insecure–disorganized, respectively [24]. Although such a specific association has not been observed in dog-owner relationships, attachment security still seem to be associated with the caregiver sensitivity to the dog’s needs in time of distress [21].

In both human and non-human animals the quality of the care received from the caregiver has been shown to affect an individual’s ability to use the attachment figure as a buffer against stress [25], leading to a dysregulation of the physiological response of both the autonomous nervous system and the hypotalamus-pituitary-axis to acute stressors [26]. For instance, children with insecure attachment patterns show higher cortisol reactivity during the SSP [27,28,29], as well as higher heart rate [28,30] and salivary alpha amylase [31], suggesting a greater activation of the sympathetic nervous system. Similarly, in adult humans, insecure attachment has been linked to primary hypertension [32], which may be predicted, among others, by parental warmth [33] and physiological reactivity to acute stress at young age [34].

With regard to dogs, there is evidence that the quality of the relationship with the owner may affect their levels of physiological indicators of both acute and chronic stress. For instance, dogs considered as “meaningful companions” or “social partners” by their owners have lower morning cortisol levels [21,35,36] Similarly, higher scores in Monash Dog-Owner Relationship (M-DORS) [36] items indicative of greater emotional closeness are correlated to lower salivary cortisol levels [37]. On the contrary, higher scores in the perceived emotional and financial costs of caring for the dog are associated with higher hair cortisol levels [38].

However, there are only a few studies that assessed dog’s physiological parameters of stress during the specific context of the SSP [22,39,40,41,42,43] and, among these, only Schöberl et al.’s study [22] correlated cortisol reactivity to insecure attachment towards the owner. Furthermore, to the best of our knowledge, there are no studies that attempted to correlate physiological indicators of chronic stress with different patterns of attachment.

In light of the scientific evidence that links attachment insecurity to acute and chronic stress in humans, as well as the scarcity of studies that used the attachment construct to explain altered physiological responses to stressful stimuli in dogs, the current study aimed at: (1) investigating possible differences in parameters indicative of sympathetic activation (i.e., heart rate, respiratory rate, blood pressure, and rectal temperature) in response to the acute stress generated by the SSP, between securely and insecurely attached dog; (2) assessing whether secure and insecure dogs show differences in cortisol reactivity during the SSP; (3) assessing a possible association between dog attachment insecurity and chronic stress, by measuring hair cortisol concentrations.

## 2. Materials and Methods

This study obtained approval from the Committee on Bioethics of the University of Pisa, Italy (review no. 29/2021) in relation to the involvement of humans, as well as a favourable opinion from the Animal Welfare Review Board of the University of Pisa (review no. 31/2021) in relation to the involvement of dogs. Owner’s informed consent and authorization to video record and use data for research purposes were obtained before each test.

### 2.1. Subjects

Experimental subjects were 20 dogs who participated with their owners. These 20 individuals were selected from a larger sample of dogs, who underwent a SSP used for attachment style classification (see paragraph 2.4) and behavioural analysis, as well as physiological sampling. These specific samples were built in order to have two matched groups, one of 10 dogs classified as securely attached to their owners, and one of 10 dogs classified as insecurely attached to their owners. Subjects were selected so that the two groups would have similar characteristics in terms of dogs’ age, sex, size and time of saliva sampling. Owners’ characteristics were also similar for the two groups, as reported in the following paragraph. Characteristics of each dog and their respective owner are reported in Table 1.

#### 2.1.1. Demographics of Secure Dogs and Their Owners

Dogs in the secure group were seven females and three males, five of which were neutered. Their age ranged from 3 to 10 years (mean = 5.30, S.E. = 0.83). Half of them were medium-, three were large- and two were small–sized dogs. Most of them were pure-breed (*n* = 7), and did not live with other household dogs (*n* = 6). For seven dogs saliva was collected in late morning, whereas for the other three it was collected in the afternoon, with a total collection time range of 6 h (from 11:00 a.m. to 5:00 p.m.), for the T0 sample.

Eight dogs had been adopted/acquired within the sixth month of age, one between 6 months and 1 year, and one between 2 and 8 years. Seven of them came from either a professional or an amateur breeder, two came from a shelter and one was born in the house of the current owner. At the time of the test, dogs had been living with their owners for 2 to 9 years (mean = 4.40, S.E. = 0.67).

Owners of secure dogs consisted of six women and four men between 19 and 59 years old (mean = 33.80, S.E. = 4.67). Most of them (*n* = 6) had a high-school diploma, while the rest had either a graduate or a post-graduate degree. Four were undergraduate students in animal-related courses (e.g., veterinary sciences, biology, animal breeding and productions, etc.), five worked jobs involving animals, and one was unemployed. Half of the owners had their first dog when they were younger than 10 years old, four when they were 10 to 20 years old and only one after that age. For three owners this was their first experience with a dog, while the rest had lived with 1 to 10 dogs, prior to the current one.

#### 2.1.2. Demographics of Insecure Dogs and Their Owners

Dogs in the insecure group were six females and four males (four neutered and six intact), ranging from 2 to 7 years (mean = 4.50, S.E. = 0.52). Half of them were large-, three were medium- and two were small–sized dogs. The majority of them were pure-breed (*n* = 6), and lived with at least another household dog (*n* = 8). Just like for the secure group, saliva was obtained in the morning from seven dogs, and in the afternoon from the remaining three, with a total collection time range of 5 h (from 11:00 a.m. to 4:00 p.m.) for the T0 sample.

Nine dogs had been adopted/acquired within the sixth month of age and only one between 1 and 2 years. Five dogs were acquired from either a professional or an amateur breeder, three were adopted from a shelter and two were born in the house of the current owner. At the time of the test, dogs had been living with their owners for 2 to 7 years (mean = 4.30, S.E. = 0.58).

Owners of insecure dogs were eight women and two men, their age ranging between 19 and 61 years (mean = 38.70, S.E. = 5.17). Three of them had either a middle school or a high-school diploma, while the remaining seven had either a graduate or a post-graduate degree. Four were undergraduate students in animal-related courses, three worked jobs involving animals, and three different professions that did not involve animals. Four owners had their first dog when they were younger than 10 years old, four when they were 10 to 20 years old and two after that age. Only for one owner this was the first experience with a dog, while the rest had lived with 1 to 8 dogs, before the current one.

### 2.2. Experimental Setting

The experimental setting was a relatively bare room within the Department of Veterinary Sciences of the University of Pisa, Italy. The room (4.50 × 4.30 m) was unfamiliar to all the dogs tested and was prepared to meet the description of the original SSP setting [3], as well as the modified setting later used to specifically test dogs [9,10,14]. The room was equipped with two chairs, one for the owner and one for the stranger; three different toys (a rope, a stuffed animal, and an empty Kong^®^ (Golden, CO, USA)) placed on the floor in the middle of the room, a table to lay the leash on; a single entrance/exit door; and two video cameras to record the whole test, placed at the two opposite corners of the room.

The tests were conducted on weekdays from 11:00 a.m. to 5:00 p.m., between August 2021 and April 2022.

### 2.3. Experimental Procedure

Owners were advised not to feed their dogs and not to eat anything in the two hours prior to beginning of the experiment, in order not to alter the saliva samples. When the owners arrived with their dogs at the location of the study, a researcher proceeded to explain them the whole procedure and give them basic instructions, such as not to start interactions with the dogs, except at specific times when they had to leave or come back into the room, or when they had to comfort them in case of distress. They were then asked to collect a saliva sample from their dogs with the methods described in the following paragraph.

Afterwards, the researcher would turn on the cameras and let the pair in the experimental room for the test to begin. A different researcher, who had never met the dog, would participate in the test in the role of the stranger and guide the owner in the different phases of the procedure. This role was always played by a person of the same gender as the owner. Previous studies have used either female and male strangers, regardless of the owner’s gender [10,44,45]. Although no effect of the stranger’s gender on dog attachment behaviour was observed by Parthasarathy and Crowell-Davies [46] in the context of the SSP, there is some evidence that the owner’s gender may affect how dogs react towards strangers of different genders [47]. The test presented the same number and order of episodes of the original Ainsworth’s SSP and it is described in detail below:

Episode 1: Owner and dog (2 min). The dog was let free to move and explore the room, while the owner was asked to sit on the designated chair.

Episode 2: Owner, stranger, and dog (3 min). The stranger entered the room. They had to stand by the door for 1 min. For the first 30 they could not start the interaction with the dog, but could respond to the interaction started by the dog, with the same level of intensity (e.g., if the dog gazed at them they could gaze back, but could not call or touch the dog). For the following 30 s, they were asked to greet the dog as if they had just entered the room. Then, they had to sit on the chair for 1 min and, again, could not initiate interactions with the dog. In the final minute, the stranger had to stimulate the dog to play with the toys, starting from the toy the dog liked less to the one they liked the most. If the dog was engaged by the play attempt with the first toy, the stranger had to go back to the chair, otherwise they had to move to the next toy, and so on.

Episode 3: Stranger and dog (3 min). The owner left the experimental room and waited for their time to return, in another room, nearby. For the first 2 min the stranger remained seated, without initiating interactions with the dog. During the third minute the stranger stimulated the dog to play with the toys, following the same procedure as in the previous episode.

Episode 4: Owner and dog (3 min). The owner returned to the experimental room and, at the same time, the stranger left. The owner followed the same protocol as the stranger in episode 2.

Episode 5: Dog alone (2 min). The owner left the room and the dog remained alone. If the dog displayed signs of intense distress, the stranger would anticipate the entrance.

Episode 6: Stranger and dog (3 min). The stranger entered the room and followed the same protocol as in episode 2.

Episode 7: Owner and dog (3 min). Same procedure as in episode 4.

As soon as the test was over, the owner was asked to remain seated in the experimental room with their dog. After 7 min the two researchers entered the room to collect another saliva sample from the dog. Then, the owner was asked to help holding the dog in place while one of the researchers performed the following physiological measurements: blood pressure, heart rate, respiratory rate and rectal temperature. Finally, a hair sample from the medial side of the hind limb was collected using an electric razor or scissors. The procedures followed to collect physiological data are described in detail in the following paragraph.

When all measurements were completed the dogs were given food treats and/or engaged in free play, depending on their preferences and their owner’s will. After testing each dog, the experimental room’s floor and chairs were cleaned using a non-toxic, weakly scented disinfectant.

### 2.4. Collection Procedure for the Dog’s Physiological Parameters

#### 2.4.1. Saliva

Saliva samples were collected immediately before (T0) and after (T1) the SSP with Salivette^®^ (Sarstedt, Rommelsdorft, Germany) swabs. Since there is evidence of a circadian rhythm in cortisol secretion in dogs [48] Fare clic o toccare qui per immettere il testo.we carefully matched the secure and the insecure groups in terms of saliva sampling time.

T0 samples were collected in an indoor waiting area outside the test room. The researcher explained the owner how to collect saliva from their dog. 

Owners were instructed to insert the swab gently under the tongue and inside the cheek pouches of the dog for 90 s, according to the methodology described in previous studies [22,49].

T1 samples were collected in the test room after 7 min from the end of test. The procedure was the same as in T0 except that, this time, sampling was performed by the researcher, while the owner was asked to help holding the dog in position, in order to collect additional data on their interactive behaviour towards the dog. The timing of the T1 sample was chosen in accordance with those reported in previous studies to determine salivary cortisol concentrations after a stressful event [39,50,51]. In our case, the sampling procedure had to be carried out 15 min after the beginning of episode 5 of the SSP (separation from the owner). All samples were immediately centrifugated and refrigerated at −20 °C. They were stocked at this temperature in the Etovet laboratory of the Department of Veterinary Sciences-University of Pisa (Italy), until they were analysed for cortisol quantification by Salimetrics Cortisol Enzyme Immunoassay Kit^®^ (Salimetrics, Segrate, Italy).

#### 2.4.2. Blood Pressure, Heart Rate, Respiratory Rate and Rectal Temperature

For each dog, measurements of blood pressure, heart rate, respiratory rate and rectal temperature were performed only once, after the completion of the SSP and after the saliva sampling at T1. Therefore, they started approximately 9 min after the end of the SSP. None of these parameters was measured before the test, in order to avoid a level of manipulation that may have affected the dog’s behaviour and physiology during the SSP.

Blood pressure and heart rate were collected simultaneously using an oscillometer (Suntech Vet30 Veterinary Monitor, Suntech Medical, Morrisville, NC, USA). The researcher applied the appropriate cuff of the oscillometer to the tail of the dog, so that blood pressure and heart rate values were obtained from the coccygeal artery. In the meanwhile, the owner was asked to hold the dog either in a recumbent or a standing position, depending on the dog’s presumed preference. Previous studies found no significant difference in oscillometric measurements of mean arterial pressure between standing and recumbent dogs [52], and the latter posture may be more stressful to maintain for some individuals. Measurements were performed for three consecutive times.

Respiratory rate was assessed by visual inspection for 30 s and the values observed were doubled in order to obtain the number of respiratory acts per minute. When breathing was too fast to be assessed, it was reported as tachypnea.

Rectal temperature was measured through a common flexible digital thermometer. Temperature measurement was performed only once.

#### 2.4.3. Hair

Hair samples were collected from dogs after the SSP test and after all the other physiological measurements were performed. The researcher used an electric razor to trim the dog’s hair from the ischiatic region, while the owners would hold the dog in position. In case the dog was evidently fearful of the electronic razor, hair was trimmed using scissors, as close as possible to the dog’s skin. All samples were wrapped in a piece of paper and stocked in a dry and dark place far from the light [53] in the Etovet laboratory at the Department of Veterinary Sciences-University of Pisa (Italy). Before extraction, the length of all hair samples was standardized cutting hair tips exceeding 2 cm. Cortisol was extracted from the matrix by using the same protocol reported in Mariti et al. [53] except for the washing procedure, which was performed using isopropanolol instead of methanol, and the use of the homogeneizer set on 6 cycles at 4350 rpm for 30 s to ground the sample to fine powder, instead of using scissors or a razor. The Salimetrics Cortisol (Expanded Range High Sensitivity) EIA Enzyme Immunoassay kit^®^ for salivary cortisol was used for the analysis [53].

### 2.5. Attachment Style Classification

Each SSP was videoed and analysed later for dogs’ behaviour in order to classify each dog using the four attachment-style scheme commonly used in children [20,54,55]. Hence, they could be classified as secure, insecure ambivalent, insecure avoidant or disorganized, based on the behavioural descriptions reported by Riggio et al. [56]. Two researchers familiar with dog attachment classification independently classified the experimental subjects. In case of disagreement, the dog’s attachment style was re-evaluated by both researchers, after a thorough discussion. Consensus was reached in all cases. Because of the small sample size, dogs showing an ambivalent, an avoidant or a disorganized pattern were combined into a single insecure group, as often seen in previous attachment studies [21,22,23].

### 2.6. Statistical Analysis

Statistical analysis was performed using GraphPad PRISM 9.0.0. Shapiro–Wilk test was performed to check the variables for normality. Independent samples T-test was used to investigate possible differences in heart rate and rectal temperature between securely and insecurely attached dogs. Mann–Whitney U test was used to assess possible differences in mean arterial pressure and hair cortisol concentrations between securely and insecurely attached dogs. Because we were not always able to visually assess the number of breaths in dogs with very high respiratory rate, we grouped them in either “normal”, if less than 35 respiratory acts per minute were observed or, otherwise, “tachypnea”. These data were analysed using a two-sided Fisher’s exact test to assess possible differences in respiratory rate between dog with secure and insecure attachment patterns. A repeated measures (RM) ANOVA with secure vs. insecure attachment style as fixed factor and time (T0 vs. T1) as within-subject factor was used to analyse dogs’ salivary cortisol concentrations. Post hoc analysis was performed using Šídák’s multiple comparisons test.

## 3. Results

**Figure 1:**
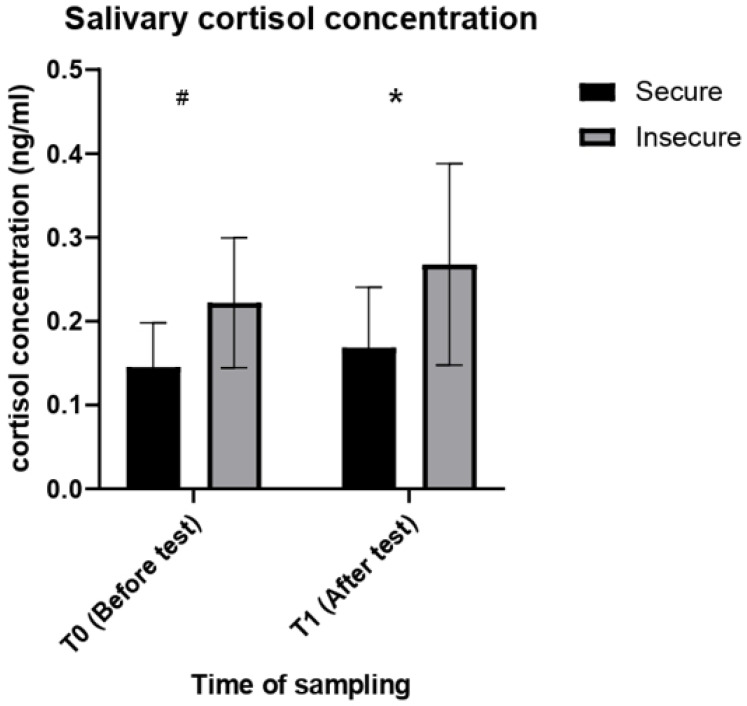
Salivary cortisol concentrations in secure and insecure dogs before (T0) and after the SSP (T1). * = *p* < 0.05. # = *p* < 0.1.

Descriptive statistics of all the physiological variables analysed are summarised in Table 2. The RM ANOVA revealed a main effect of the attachment style on the concentrations of salivary cortisol (F (1, 18) = 8.830, *p* = 0.008) in the dogs tested. According to the results of the post hoc analysis, insecure dogs had significant higher salivary cortisol concentrations than secure dogs at T1 (*p* = 0.024), but only a weak, non-significant trend towards higher cortisol concentrations at T0 (*p* = 0.099) (Figure 1). No significant effect of time of sampling (T0 vs. T1) on salivary cortisol concentrations was found (F (1, 18) = 2.176, *p* = 0.157).

Furthermore, no significant difference was found in hair cortisol concentrations between dogs classified as secure and those classified as insecure (U = 41.00, Z = −0.68, *p* = 0.496).

Among the physiological parameters measured after the test, mean blood pressure (t (18) = −0.07, *p* = 0.941), respiratory rate (*p* = 0.303, Fisher’s exact test) and rectal temperature (t (17) = −1.55, *p* = 0.140) did not significantly differ between secure and insecure dogs. Heart rate showed a non-significant trend towards higher values in insecure compared to secure dogs (t (18) = −1.88, *p* = 0.077).

## 4. Discussion

This study explored whether adult pet dogs classified as either securely or insecurely attached to their owners show a different physiological response to stress. Although not statistically significant, the overall increase in salivary cortisol concentrations during the SSP is worth further discussion, as it seems to suggest that the experimental procedure was effectively perceived as moderately stressful by both secure and insecure dogs. This should not be surprising since the SSP is specifically designed to progressively increase the level of stress in the individuals tested [20]. Furthermore, dogs involved in the SSP are commonly reported to show behavioural signs of stress during the procedure (e.g., aimless wandering, vocalizations, escape attempts, scratching the door) [8,12,56,57]. Nonetheless, the increasing cortisol trend found in this study reflects that reported by Mongillo et al., [39] for aged dogs, but differs from the decreasing trend observed by Schöberl et al. [22] and Ryan et al. [40]. These conflicting findings may be due to methodological reasons. Firstly, Ryan et al. [40] only tested dogs that were classified as securely attached, which possibly had a reduced activation of the HPA axis during the test [22,40]. Secondly, while we did ask the owners to avoid stressful and exciting stimuli before the test, we did not apply a standardized procedure, nor is one described by Schöberl et al. [22] and Ryan et al. [40] in their studies. Therefore, environmental and social stimuli encountered prior to the first saliva sample may also have led to different results. Lastly, in both Schöberl et al. [22] and Ryan et al. [40], a decrease in cortisol concentrations was observed between the sample taken before the test and the one taken immediately after the test. Instead, in our study, the post-test sample was taken 7 min after the end of the SSP, so that it would reflect the cortisol response of the dog when left completely alone. In fact, in Schöberl et al. [22], at the time of the sample taken 15 min after the SSP, cortisol concentrations had increased again. Hence, it is possible that sampling saliva immediately after a 20 min long test in which the level of stress is supposed to increase progressively, does not provide a sufficient time interval to assess the peak of the dog’s cortisol response to the procedure, which is likely to occur during episode 5, the most stressful episode of the SSP, when the caregiver leaves the dog alone in the room [51].

In the current study, we found that cortisol concentrations were significantly higher in insecure dogs at T1 and higher on average, although not significantly, in the same group at T0. Overall, these findings confirm those reported in Schöberl et al.’s [22] study, the only previous study to have investigated the dog cortisol response during the SSP in relation to attachment insecurity. However, while Schöberl et al. [22] did find that cortisol reactivity to the SSP was different between secure and insecure dogs, they did not focus on whether absolute cortisol concentrations between the two groups were different before or after the test. In contrast, in our study, insecure dogs began the SSP with slightly higher cortisol concentrations, suggesting they had a different physiological response to stressful stimuli encountered prior to arriving at the test location (likely not differing for secure and insecure dogs) and/or a higher basal level of stress, as occurs in insecurely attached humans [58,59]. This finding requires further investigation in terms of both confirmation with larger samples and possible explanations of the relationship between attachment and response to stressors of different nature, in dogs. Furthermore, the fact that cortisol concentrations, although higher in insecure dogs at both T0 and T1, reached a statistically significant difference only after the test, seems to suggest a different trend in cortisol reactivity to the SSP, in relation to attachment insecurity [22]. Considered the timing of our post-test sample, the cortisol levels reported are likely to reflect the higher level of stress of insecure dogs in response to the separation from the owner, confirming previous observations on both insecure dogs [56] and human infants [60].

Among those physiological indicators of sympathetic activation that were measured after the end of the test (heart rate, blood pressure, respiratory rate, rectal temperature), only heart rate tended to be higher in insecurely attached dogs, although the difference did not reach statistical significance. Previous studies have used heart rate or other parameters of cardiac activation to assess dogs’ physiological stress response during the SPP [41,42,43]. However, to the best of our knowledge, this is the first study that attempted to correlate physiological indicators of sympathetic activation to the pattern of dog attachment towards the owner. On the other hand, in human literature, previous studies report an association between infants’ attachment insecurity and altered cardiac activity during social stress [61]. For instance, Sroufe and Water [30] found that only securely attached children showed a rapid heart rate decrease after reuniting with the caregiver, while Spangler and Grossman [28] found a particularly high increase in heart rate in disorganized infants involved in a SSP. However, most of these studies recorded cardiac parameters throughout the procedure, while we collected these data at a single time point, approximately 9 min after the end of the test. Therefore, while our findings still suggest that there may be an association between dogs’ cardiac activation during a stressful event and attachment insecurity, the former may not reflect the dogs’ physiological response to the SSP, rather to the measurement itself. Since the sympathetic nervous system is known to rapidly, but briefly affect the bodily response to a stressor, future studies aimed at assessing dogs’ stress response during the SSP should either anticipate the physiological measurements or use devices that allow for continuous data recordings. Although previous studies have used such devices [41,42,43], the preparation of the dog’s body is often described as a relatively invasive procedure [62], which may affect both the behavioural and the physiological response of the experimental subjects during the SPP. Furthermore, their ability to correctly record data may be compromised when applied to even moderately active and aroused individuals [63]. In any case, possible associations between cardiac activity and attachment styles in dogs have not been evaluated, so far.

Interestingly, although no statistical difference was found in the percentage of tachypneic individuals in the secure and the insecure group, in the latter all but one dog displayed tachypnea. Therefore, it may be interesting to further investigate this parameter in future studies with larger samples.

In the present study, no difference in hair cortisol concentrations was found between securely and insecurely attached dogs. Although there is some evidence that aspects of the dog-owner relationship, such as positive interactions [64,65] or the owner’s reduced perception of the costs of caring for the dog [38] may be associated with lower hair cortisol concentrations, this is the first study to investigate a possible association of the latter with dog attachment insecurity. In human literature, attachment insecurity seem to be a predictor of chronic stress-related health problems, such as cardiovascular diseases [66] and inflammatory-related illnesses [67]. Furthermore, insecure attachment styles have been associated with the development of emotional and psychological disorders in both children and adult individuals [68,69,70], which in turn may be associated with altered cortisol levels [71]. Despite that, there is a surprisingly low number of studies investigating hair cortisol concentrations in relation to attachment insecurity, in humans. While there is evidence that a sub-optimal caregiving behaviour from a parent may be associated with higher hair cortisol levels in the child [72,73,74], there seems to be no direct association between the latter and attachment insecurity neither in young [75] nor in adult individuals [76]. In this pilot study, we did not assess the owner’s behaviour during the SSP. However, as it appears to be in humans, it is possible that the caregiver’s behaviour, rather than the corresponding attachment style of the dog, may better explain hair cortisol concentrations in the latter. It may be interesting to investigate this hypothesis in future studies.

An obvious limitation of this study is the small sample size. For this reason, we could not assess possible differences in physiological indicators of stress between avoidant, ambivalent and disorganized subjects. On the contrary, we decided to combine them into a broader insecure group to test against securely attached individuals. While this decision is neither conceptually nor methodologically incorrect, it does not allow to bring the specific characteristics of each insecure attachment style to light, as already done in human psychology [61]. Moreover, larger samples may allow future studies to reliably test for confounding environmental and social variables that may affect the measurement of physiological indicators of stress in dogs (see Chmelíková et al. [77] for a review on salivary cortisol; see Heimbürge [78] and Masercova [79] for a review on hair cortisol) or the quality of dog attachment, such as the presence of other dogs in the household. Previous studies have shown that dogs may form affective bonds with conspecifics living in the same household [5,8,23,57,80] and that the quality of this bond may affect the relationship with their owners [81]. Our study was not designed to test the effect of this or other variables on dog attachment, but they should be investigated in future studies with larger samples.

Our sample was also too small to assess whether dog breed affected the level of stress of the dogs during the SSP. In this regard, a recent study by Lenkei et al. [82] found no difference in anxiety behaviour between cooperative and non-cooperative working dogs involved in a SSP, suggesting that the procedure was equally stressful for all subjects, regardless of their breed selection. However, previous studies suggest that some breeds may be more negatively affected than others by the separation from the owner [83,84], which is the greatest source of stress for dogs during the SSP. Furthermore, fear and anxiety, in general, as well as attention seeking behaviours seem to have a higher prevalence in toy breeds [85], possibly affecting their level of stress in the specific context of this experimental procedure. Not less importantly, specific breeds may be more often associated with owners with attitudes and personality traits that affect their ability to be sensitive and responsive to their dogs’ needs and ultimately function as a source of emotional support in times of distress. For instance, owners of dogs belonging to breeds considered “aggressive” (e.g., German shepherd, Rottweiler) or “vicious” (e.g., Dobermann, Pit Bulls) are more likely to show personality traits associated with psychoticism (e.g., propensity to have psychotic episodes and a tendency towards being angry, hostile and aggressive) or psychopathic tendencies (e.g., carelessness and selfishness), respectively [86,87].

It is therefore conceivable to think that factors directly or indirectly related to breed may have an effect on dogs’ behavioural and physiological measures of stress during the SSP. However, this assumption requires a deeper investigation on larger samples of dog breeds characterized by different functional breed selections.

Although the findings from the current study should be regarded as preliminary, they provide interesting conceptual and methodological hints for future dog-owner attachment research.

## 5. Conclusions

Despite the overall visible increase, no significant difference could be found in salivary cortisol concentrations between T0 and T1 for neither secure nor insecure dogs. Insecure dogs showed a non-significant trend towards higher salivary cortisol concentrations compared to secure dogs even before the test. This may suggest that the former may have had a different physiological stress response to stimuli encountered prior to the beginning of the SSP. Nevertheless, the finding that insecure dogs showed significantly higher salivary cortisol concentrations only after the SSP, seems to point towards a more pronounced cortisol response to the stress induced by the experimental procedure, as observed in previous studies [22]. Among the indicators of sympathetic activation measured after the test, only heart rate tended to be higher, although not significantly, in insecure dogs, suggesting a possible, more pronounced cardiac activation of these dogs in response to stress. Whether the latter was represented by the SSP or the measurement itself should be further investigated in future studies by modifying the timing of physiological data collection.

Lastly, this was the first study to attempt to correlate attachment insecurity with hormonal parameter of chronic stress, i.e., hair cortisol. In this regard, no differences were found between insecurely and securely attached dogs. These findings should be considered preliminary and larger samples may be needed to better understand the link between attachment insecurity and physiological response to stress in dogs.

## Figures and Tables

**Table 1:** Basic demographic characteristics of each dog-owner dyad and dog attachment classification.

Dog	Breed	Size	Sex	Age (Years)	Owner’s Gender	Owner’s Age	Attachment Style
1	Boxer	Large	Female	6	Female	27	Insecure
2	Mixbreed	Large	Male	4	Female	27	Insecure
3	Australian Shepherd	Medium	Female	2	Female	25	Insecure
4	Maltese	Small	Female (s)	5	Female	59	Insecure
5	Labrador Retriever	Large	Female (s)	7	Female	59	Insecure
6	Jack Russell Terrier	Small	Female	5	Female	50	Insecure
7	Mixbreed	Large	Male (n)	6	Male	26	Insecure
8	Mixbreed	Medium	Male (n)	4	Female	61	Insecure
9	Mixbreed	Large	Male (n)	2	Male	22	Insecure
10	Lagotto Romagnolo	Medium	Female (s)	4	Female	31	Insecure
11	Bernese Mountain Dog	Large	Male	3	Male	22	Secure
12	Labrador Retriever	Medium	Female (s)	10	Female	23	Secure
13	Golden Retriever	Large	Female (s)	6	Female	49	Secure
14	Mixbreed	Small	Male	7	Female	20	Secure
15	Australian Shepherd	Medium	Female (s)	4	Male	48	Secure
16	Cocker Spaniel	Medium	Female	5	Female	59	Secure
17	Cavalier King Charles Spaniel	Small	Female	3	Male	45	Secure
18	Labrador Retriever	Medium	Female (s)	3	Female	19	Secure
19	Mixbreed	Medium	Female (s)	9	Female	27	Secure
20	Mixbreed	Large	Male	3	Male	26	Secure

(s) = spayed, (n) = neutered.

**Table 2:** Descriptive statistics of the physiological data in secure and insecure dogs.

Physiological Variables	Secure Dogs		Insecure Dogs	
	**Min–Max**	**Mean (S.E.)**	**Min–Max**	**Mean (S.E.)**
Salivary cortisol at T0 (ng/mL)	0.09–0.27	0.14 (0.01)	0.14–0.40	0.22 (0.24)
Salivary cortisol at T1 (ng/mL)	0.09–0.35	0.16 (0.02)	0.11–0.54	0.26 (0.03)
Hair cortisol (pg/mg)	3.81–11.07	6.55 (0.77)	2.34–20.93	7.10 (1.84)
Heart rate (pulse/min)	73–120	98.10 (5.24)	82–144	114.20 (6.78)
Mean blood pressure (mmHg)	77–119	88.60 (4.39)	67–104	89.10 (3.86)
Rectal temperature (°Celsius)	38.0–39.1	38.76 (0.10)	38.3–40.1	39.1 (0.21)
	**Normopneic (%)**	**Tachypneic (%)**	**Normopneic (%)**	**Tachypneic (%)**
Respiratory rate	4 (40)	6 (60)	1 (10)	9 (90)

## Data Availability

Data are available on request from the corresponding author.
